# Of aquifers and agency

**DOI:** 10.1007/s10040-026-03059-6

**Published:** 2026-03-18

**Authors:** Adrian Healy

**Affiliations:** School of Geography and Planning, Cardiff University, Cardiff, UK

**Keywords:** Agency, Aquifers, Socio-hydrogeology, More-than-human, Groundwater

## Abstract

In this paper, I consider the role of agency in complex and adaptive socio-hydrogeological systems, particularly the extent to which aquifers may, themselves, exhibit agency. I argue for a characterisation that appreciates aquifers as part of a complex, adaptive and co-evolving more-than-human landscape with the capability to exert agency through performative intra-action. Acknowledging this agency of aquifers within socio-hydrogeological systems offers fresh insights regarding the emergence of water-sensitive urban/rural development pathways and opens up novel interdisciplinary research agendas.

## RESUME

Dans cet essai, j'examine le rôle de l'agentivité au sein des systèmes socio-hydrogéologiques complexes et adaptatifs, et plus particulièrement la mesure dans laquelle les aquifères peuvent, eux-mêmes, manifester une agentivité. Je défends une caractérisation qui appréhende les aquifères comme faisant partie d'un paysage qu’humain complexe, adaptatif et co-évolutif, capable d'exercer une agentivité par le biais d'interactions performatives. La reconnaissance de cette agentivité des aquifères au sein des systèmes socio-hydrogéologiques offre de nouvelles perspectives sur l'émergence de modèles de développement urbain/rural sensibles à l'eau et ouvre de nouvelles perspectives de recherche interdisciplinaire.

## RESUMEN

En este ensayo considero el papel de la gestión en sistemas socio-hidrogeológicos complejos y adaptativos, especialmente en la medida en que los acuíferos pueden, por sí mismos, mostrar dicha gestión. Defiendo una caracterización que valore los acuíferos como parte de un paisaje complejo, adaptativo y coevolutivo más que humano, con la gestión a través de la intra-acción performativa. Reconocer esta gestión de acuíferos dentro de los sistemas sociohidrogeológicos ofrece nuevas perspectivas sobre la aparición de vías de desarrollo urbano/rural sensibles al agua y abre nuevas agendas de investigación interdisciplinar.

## Abstract (zh)

本文探讨能动性在复杂适应性社会—水文地质系统中的作用，尤其关注含水层自身在何种程度上可能表现出能动性。本文主张采用一种表征框架，将含水层理解为复杂、适应性且协同演化的“多于人类”景观的一部分，并认为含水层能够通过表述性行为的内作用发挥能动性。在社会—水文地质系统中承认含水层的这种能动性，可为水敏感型城市/乡村发展路径的机制提供新的认识，并开启新的跨学科研究议程。

## RESUMO

Neste ensaio, considera-se o papel da ação em sistemas socio-hidrogeológicos complexos e adaptativos, particularmente a extensão em que os aquíferos podem, por si só, escolher qual ação tomar. Defendo uma caracterização que valorize os aquíferos como parte de um cenário complexo, adaptativo e coevolutivo mais que humano, com capacidade de exercer ação por meio da intra-ação performativa. Reconhecer essa ação de aquíferos dentro dos sistemas socio-hidrogeológicos oferece novas percepções sobre o surgimento de caminhos de desenvolvimento urbano/rural sensíveis à água e abre novas agendas de pesquisa interdisciplinar.

## Introduction

In a recent paper, Blake et al. (2023) put forward the suggestion that an aquifer system, and its associated dependents, should be viewed as the client in hydrogeological investigations. This subtle but nonetheless significant ontological shift, where an aquifer is not merely the subject of study or object of interest but is considered a distinct actor in socio-hydrogeological systems, raises a profound question as to whether an aquifer might then be considered to have agency. By agency, I mean the capability to act, to influence other actors, and, in the case of an aquifer, to shape its environs and corresponding socio-hydrogeological systems.

Physically, an aquifer is typically described as ‘a formation, group of formations, or part of a formation that yields water of suitable quality to wells or springs in economically usable amounts’ (Sharp 2023), capturing both the material dimensions (materiality) of aquifers and their conception in terms of use-value. Moving beyond this, the hydrosocial turn in water scholarship across the natural and social sciences begins to conceptualise water as simultaneously social and hydrological (Alba et al. 2025; Cuthbert 2025). Groundwater, Huggins et al. (2023) argue, connects social, economic, ecological, and Earth systems, as part of broader complex and adaptive socio-hydrogeological territories. In a similar vein, aquifers can also be seen as part of a ‘hydro-geo-social choreography of everyday life’ (Ballestero 2023a). This choreography emerges through the interaction of human and non-human forces, that collaborate in the making of urban and rural spaces over time (Powis 2021; Hadfield-Hill and Zara 2019).

Following Birkinshaw’s (2022) example of groundwater, the recentring of aquifers as actors in a connected world raises critical questions of the relative agency of people, technology, ecology and hydrogeology. It provides a vehicle for reconsidering pathways of change, adaptation and transformation and for recognising the power that material and immaterial properties of matter can exert. Traditionally, agency has largely been described in humancentric terms—such as a feeling of control over actions and their consequences (Moore 2016). However, there is a broader literature that challenges this human exceptionalism, which this paper begins to draw upon.

Across that literature writers variously refer to actors, actants, agents and bodies. Each has a distinct meaning in the writings in which they are set, but commonly refer to entities representing people, groups, organisations and other animate and inanimate beings/things. In this paper, I preference the term actor but recognise this may obscure some of the more nuanced meanings of different terminologies.

Recognising the potential agency of aquifers as actors moves beyond traditional perspectives that see aquifers as objects to be studied, monitored and managed. Fundamentally, it seeks to understand the role of the aquifer as an agent of change, or stability, within a wider socio-hydrogeological system with the power to influence the actions of others and their capability to act. Considering the agentic power of aquifers opens up questions regarding the extent to which aquifers exert influence over the distributional consequences of perturbations in the socio-hydrogeological system and the normative implications for emerging concepts of justice.

## Conventional perspectives on agency

Across the social sciences the concept of agency holds that humans have the capability to shape the circumstances in which they live, through the resources at their disposal, the choices they make and the actions that they take (Emirbayer and Misch 1998; Barker and Jane 2016; Haggard and Eitam 2015). It highlights the power of individuals to shape their own lives and the social world around them, rather than being solely determined by external structures (Sulkunen 2009). As such, agency has a productive power, which can reproduce existing circumstances or create new contexts (Ferrero 2022). Typically, academic literatures characterise agency by the potential consequences of action (or inaction) by the actors involved. This may take the form of allowing/enabling change or resisting/blocking behaviours. As such, agency can be seen as a creative force for betterment (producing something new or transformative); as maintaining or reproducing an existing state of affairs or, potentially, as being (self)-destructive (Pearson 2015; Jolly et al. 2020; Baumeister 2024).

Academic opinion divides as to whether human agency is intentional and purposeful, targeted towards the achievement of identified goals (Bandura 2000; Moore 2016; Grillitsch and Sotarauta 2020), or can be instinctive, unintentional, habitual and unthinking (Latour 2005). Indeed, Latour (2005) argues that intention is an unhelpful construct, whilst Barad (2007) highlights how difficult it can be to cut through the complex interplay between different influences to discern true intent and purpose, particularly when only the individual can know their own, underlying, motivations, with onlookers having to infer these through observation.

Whilst agency is commonly associated with the individual, an important extension to the idea of human agency is that actors do not have to act independently. Individual actions can have power but change at the societal level tends to occur when groups intentionally or unintentionally coalesce (Ludwig 2016). Collective agency can emerge organically, through uncoordinated collective action, and through the emergence of institutional structures that promote collective voice and action (Shariff 2018). In these circumstances agency is constructed through the discursive interaction of actors shaped by reflexive and recursive practices (Toivonen 2022). Similarly, in other circumstances actors may be content to, implicitly or explicitly, transfer their agency to others to act on their behalf. Latour extends this concept to stress that humans never act in isolation, human agency he argues is co-produced by multiple forces acting in concert (Latour 2005).

To enact agency it is not sufficient to simply wish to act, as individuals differ in their opportunities or capabilities to do so (Sen 2009). Differential access to resources, varying skill sets, differences in social status, cultural or political reach may all influence both our capacities to act and the choices that we make. As Sen avers, for an individual or group, the ‘freedom of agency … is qualified and constrained by the social, political and economic opportunities that are available to us’ (1999). In making this claim, Sen follows a long tradition which centres agency within the co-constituted confines of social structures, which enable or constrain actors and their decisions (Giddens 1979; Barker and Jane 2016).

Disentangling the relative influence of agency versus structure in explaining the dynamics of change has been an ongoing field of research (Hay 2009; Sotarauta et al. 2022). As social structures are relational constructs, experienced differentially by different actors, then actors in superficially similar material circumstances may act in wholly dissimilar ways (Coole 2005). Equally, as structures are co-constituted with agency, they are not wholly deterministic but can be formed, shaped and reshaped by the interventions of human actors exerting agency (Jessop 2005; Sotarauta et al. 2022). All action has a generative capacity, not only through its own effect but also through its representation by others (Sulkunen 2009). For Sulkunen (2009) this more generative agency is crucial in moving from what he describes as a sociology of action to a sociology of the actor and its environment.

Furthermore, agency is not a constant but is spatially and temporally situated. We will all be familiar with how our own sense of our ability to shape our circumstances can vary depending on the context, the time and the space in which we are located (Grillitsch et al. 2025). Equally, the capability for agency need not be constantly exercised, it may be activated or de-activated according to setting (Sotarauta et al. 2022). Moreover, as Emirbayer and Mische (1998) emphasise, human actions are informed by our memories of past experiences alongside our perspectives of the future, and the shadow this casts on the present. It is the interplay of these temporal dimensions with our current circumstances that informs selected actions. As such, an analytical understanding of agency can only be fully realised when situated within the ‘flow of time’ (Emirbayer and Mische 1998). The notion that agency is not automatic but has to come into being suggests the presence of critical junctures for change at particular moments in time, or moments in life, which can profoundly shape the direction of actions taken (Pierson 2004; Grillitsch and Sotarauta 2020).

## Incorporating non-human agency

In response to charges of human exceptionalism, acceptance of the potential for non-human actors, such as nature, animate beings or inanimate objects, to have agency has gathered momentum in recent years (Pearson 2015). For some writers, such as Williams (2022), nature has always had agency, it is simply that the agency of humans now dominates and overpowers that of nature; to the extent that we no longer recognise it. Other writers build on the groundbreaking work of Abram (1996), and acknowledge the complex interdependencies that exist between humans, nature and technology, leading to a popularisation of the term ‘more-than-human’ (for further reading, see Haraway 2016).

A crucial element in the turn towards recognising a non-human, or more-than-human, agency is the assertion that agentic behaviour does not rely on human levels of intentionality and rationality (Latour 2005, 2014). As such, there is no reason for agency to be a human trait alone. This is not to downplay the potential for humans to act with intent, but rather, as Pearson (2015) comments: ‘The challenge is to consider how these different types of agency interact and how nonhuman actors allow, as well as foil, human activities and plans’. It is this acting through interaction that provides the context for speaking of the more-than-human, rather than focusing on the non-human alone (Lorimer and Hodgetts 2024). In nature, these interactions provoke an unpredictability, and even ‘unruliness’, in response to human intentions (Bakker 2004).

Drawing on Latour’s (2005) assertion that it is networks of human and non-human ‘actants’ that determine social networks rather than humans alone, the idea that artefacts or animate beings can influence the actions of humans has received strong attention. Echoing the groundbreaking work of de Laet and Moll (2000) who describe the agency of the Zimbabwe Bush Pump, writers such as Caronia and Mortari (2015) suggest that not only do objects mediate how human actors see and engage with the world around them but that humans and objects both participate in constructing the world we see around us and cooperate to do so. In this reading, more-than-human actors have causal agency through their relationship to human actors, by influencing their actions, responding to those actions and co-constituting further responses (Frigerio 2020). Crucially, it is not only the material properties of the more-than-human that enables agency but also immaterial dimensions (Bennett 2010), such as how aquifers are imagined by different users or cultures.

In addressing the agency of the more-than-human we also move away from dividing the world into subjects (that have agency) and objects (which do not) and allow for the generative capacities of biophysical processes (Sulkunen 2009; Bennett 2010; Bakker and Bridge 2006). By doing so, we allow for the potential that agency is a collective property that comes into being through the conjoint actions of the various actors/actants who never really operate alone (Latour 2005; Bennett 2010). Various terminologies have been coined to describe these groupings. Bennett, for example, uses terms such as a ‘congregational understanding’, a ‘confederation of human and non-human elements’, and ‘an agency of assemblages’.

Regardless of the phrasing, the key point that both Latour and Bennett stress is that agency is a dynamic and relational property that is distributed across human actors and the more-than-human in ways that are mediated by prevailing structures and exchanges that, in turn, provoke further events in a recursive process (Bennett 2010). How these events unfold reveals much about the geometries of power that shape the relationships between actors, and the consequences of their actions, across geographies and over time (Swyngedouw 2004).

## Inserting aquifers into contemporary discourses of agency

Hydrogeologists are familiar with the idea that aquifers are dynamic constructs that have power and influence. This concept of agency and vitality of matter underpins the philosophy of Jane Bennett (2010), who contends that material things have the power to influence events and human lives. Extending this concept of the power of things into hydrogeology and aquifers highlights the importance of treating aquifers as active actors, rather than as simply objects with physical properties to be understood.

Arguably, aquifers exert power through mediating access to groundwater and their affect on physio-chemical characteristics of that water. Whilst, by definition, all aquifers contain ‘usable’ or ‘useful’ quantities of water (Sharp 2023; Van der Gun 2022), individual aquifers exhibit unique and dynamic features depending on the nature of the groundwater they hold and their physical structures (Tóth 1999). Aquifers also exhibit internal complexity with sometimes divergent properties that vary by depth and across space (Guillaume et al. 2016). These varying properties, when intertwined with human–groundwater interactions, form symbiotic or co-dependent relationships that are both temporally and spatially situated (Jasechko et al. 2024; Mukherjee et al. 2024; Velis et al. 2017).

The extent to which aquifers influence the quantity, quality and distributional dynamics of groundwater flows is well-documented. They can affect land values; patterns of habitation, agricultural and industrial production; the risks of ill-health through exposure to contaminants or trace elements such as uranium, arsenic and fluoride, and can differentially influence the quantity of water accessible to different socio-economic groups (see, for example, Edwards 2016; Hornbeck and Keskin 2014; Shamsudduha et al. 2022; Williams et al. 2024). The affect of aquifers is not solely dependent on their material properties. Aquifers also exert influence through how we imagine them, ranging from Cobbing and Hillier’s (2019) critique of a ‘discourse of scarcity’ limiting groundwater investments in sub-Saharan Africa, through Bessire’s (2022) identification of aquifer ‘aporias’ in the US, to the spiritual significance and alternative worldviews (Martinez-Cruz et al. 2024) that have shaped how and where humans interact with aquifers over time.

At a material level, the multifaceted and heterogeneous nature of aquifers influences how they differentially respond to different stressors, even within relatively local geographies (MacDonald et al. 2016). As complex bodies, aquifers can surprise us, particularly when they react in ways that we did not expect based on our available knowledge (Mukherjee et al. 2024; Powis 2021; Domínguez-Guzmán et al. 2023). The extended response times of aquifers also means they carry a geochemical and hydraulic legacy of past interactions, which can outstrip human memory, yet may still influence current behaviours of the aquifer and potentially the future (Brooks et al. 2021; Cuthbert et al. 2023; Schuler et al. 2022; Sherif et al. 2019). Recognising that our contemporary experience of aquifers is both spatially and temporally situated leads Ballestero (2023b) to describe aquifers as ‘emplaced formations, with legacies that persist’. Thus, aquifers embody Emirbayer and Mische’s (1998) observation regarding the importance of the temporal dimension of agency, demonstrating its salience for the more-than-human, as well as the human.

In considering human–aquifer interactions, it is also important to reflect on the variety of concepts and measurement techniques utilised by hydrogeologists, and how particular approaches may influence our characterisation of a particular aquifer. In her influential work *Meeting the Universe Halfway* (2007), Karen Barad argues that entities, such as aquifers, do not pre-exist as fixed forms but emerge through material-discursive practices. She emphasizes that our measurement techniques and conceptual frameworks actively participate in producing what we come to understand as an ‘aquifer’, rather than merely revealing an independent reality. Equally, how we perceive an aquifer shapes our expectations of it. By example, in his consideration of aquifer depletion on the High Plains of the US, Bessire (2022) highlights how ‘the physical characteristics of an aquifer condition the social knowledge of it even while the exploitation of an aquifer system may alter its physical characteristics’.

Of course, as Willey (2016) and Ballestero (2019) caution, we should be wary of how techno-scientific notions shape what we perceive. Increasingly, authors are alert to alternative ways of knowing, of considering water not solely as a resource but also as a lifeforce or spiritual embodiment (Cleaver et al. 2023; Martinez-Cruz et al. 2024, see also Ray and McCormick (2023) and Parsons and Fisher (2020) for discussion of alternative worldviews of freshwaters). Heterodox perspectives of what an aquifer is, and of human–aquifer inter-relations, may influence the agentic role of the aquifer through foregrounding immaterial characteristics, such as faith in spiritual or healing properties, alongside the material properties more traditionally highlighted in western science.

Thus, in considering agency in the context of aquifers, we must be alive to the sheer variety of interactions with, and within, an aquifer and how these vary over time and by place. These interactions are multiple and individual, both material and immaterial, and are temporal, dynamic and relational. Crucially, individuals and communities will each have a particular and differential experience of these spatial and temporal dimensions, depending on their own perspective, needs and desires (Massey 2005). This concept of relational space and time is not static but is continuously being created and recreated, laying the foundations for new configurations in the future (Fuller 2024). Inserting aquifers into contemporary discourses on agency involves recognising the recursive relationship between aquifers and human society, with each exerting influence and affect, positive and negative, on the other. This may involve human actions following the lead of the aquifer (Seidl et al. 2024), or the affect of human actions on an aquifer, ranging from over-abstraction and contamination to regenerative recharge (Winter et al. 1998; Dillon and Arshad 2016; Shamsudduha et al. 2022). Recognition of the exploitative relationship that human society can exert is leading to calls for new narratives of care to guide our interactions with entities such as aquifers (Zwarteveen et al. 2024, Houart et al. 2025).

## Embedding agency in socio-hydrogeological systems

It is broadly accepted within hydrogeological scholarship that aquifers are bonded in a lively dynamic with human society, nature and groundwater to form interconnected, complex and adaptive socio-hydrogeological systems (Ballestero 2019; Huggins et al. 2023). These systems comprise several multi-scalar features that influence both the technical and material dynamics of human–aquifer interactions. This is particularly so for aquifers, where quantities, qualities and accessibility of groundwater can vary both in space and time, and can operate at very different scales to human activity (Birkinshaw 2022; Bessire 2022; Cuthbert et al. 2023; Guillaume et al. 2016).

A key feature of complex and adaptive systems, such as aquifers, is their ability to self-organise and spontaneously respond to external events (Bak 1996; Bristow and Healy 2014). Drawing on the philosophy of Spinoza, Bennett (2010) describes the bodies that constitute complex systems as ‘affective’ in that they affect or are affected by other bodies in the system. As particular components in a system react to change, they induce change in other parts of the system, which induce further responses in an ongoing cascade of change. The cumulative results of these changes are not always predictable as affect is broadly distributed and so outcomes at a system-scale are characterised as emergent (Corning 2002).

Centering aquifers within socio-hydrogeological systems opens up new perspectives on agency. At one level, agency may be located in the accumulations of actions by multiple actors and their iterative and recursive affects on others (Dinmore et al. 2023). Crucially, this includes the actions of hydrogeologists as ‘interpreters’ of an aquifer and its properties, particularly as they seek to unpack the ‘uncertainty’ that often prevails in dialogues regarding aquifers and groundwater (see Latour 2014 or Barad 2007 for discussions on the subjectivity of the researcher).

Alternatively, agency can be considered at the level of a system as a whole, recognising how interactions occur at multiple scales involving multiple actors (Grillitsch and Sotarauta 2020; Newey 2023; Ziembla et al. 2025). Taking such a systems perspective provides fresh insights into the relative agencies of people, technology and ecology and how these enable or limit action (Birkinshaw 2022). For Grillitsch and Sotarauta (2025), as for Bennett (2010), it is the distribution of agency across a system that is critical to how new possibilities can be brought into being, or actions constrained, with the affective power of actors being enhanced or inhibited through being part of a wider assemblage. This has echoes of relational concepts of power (see Honneth, in Ibsen 2023), where power is shaped and constituted by the situated relationships, both co-operative and conflictual, between actors.

Following Bennett (2010), I suggest that agency is distributed across a socio-hydrogeological system and includes both human and non-human actors, each of which are also shaped by the creative activity that occurs within them. Crucially, it is not only humans and their institutions that have active, creative power but ‘inanimate’ matter is similarly active and so able to shape humans and society, whilst being, in turn, affected by the actions of humans. This creates a ‘lively’ materiality which, when coupled with the epistemic, socially and politically constructed uncertainty of the properties of an aquifer over time (Bessire 2022; Hamilton, this volume) makes for a very dynamic and, as Birkenholtz (2015) puts it, potentially unstable, system-level configuration. As such, the agency that aquifers enact is an emergent property situated in time and place.

The dynamic complexity of spatio-temporal relations present within socio-hydrogeological systems lends weight to the idea that it is more productive to see systems as entangled states of agencies (Barad 2007) rather than simply a collection of agents or actors. Barad (2007) contends that if we take a systems perspective then we must move beyond the notion that the system is simply an aggregation of individual entities with inherent properties. In this reading, agencies do not exist as individual elements but are only distinct in relation to their mutual entanglement. Thus, agency is not a specific property or a capacity, but is a doing, an enactment or a flow (Barad 2007; Bennett 2010). Barad describes this dynamic portrayal of agency as being performative.

In Barad’s reinterpretation of agency as performative, it is neither the aquifer that causes something to happen, nor the individual or society. Rather, it is a complex set of intra-actions (the doing) that influence the emergent outcomes observed. Barad introduces the term intra-action to emphasise that in forming a relationship, bodies or actors, are no longer independent but become co-dependent and are co-constituted. This addresses the thorny question of causality in that there is no linear chain of cause–effect (unless viewed retrospectively). Barad stresses the significance of intra-action over interaction as she wishes to highlight the importance of internal, and often unseen, processes.

For Barad, different intra-actions produce different phenomena. In the case of an aquifer, we may not interact with it directly, but we all intra-act with, and co-create, the phenomena that emerges as aquifers and socio-hydrogeological systems. Barad describes her approach as one of agential realism, in that she recognises that there is a world that is independent of the human, but as soon as we engage with that world, it is no longer independent. We interpret it and shape it according to how we wish to see it and to use it. In this reading, the various datasets we have on different aquifer properties, and how these are presented, equally have agentic power, as alluded to in several papers in this Special Issue.

In proposing a performative concept of agency, Barad (2007) draws on the concept of diffraction to suggest that overlapping agencies do not merely combine but rather create contrasting patterns of interference. Where agencies align, they can create patterns of reinforcement (constructive interference) which may be positive or negative. Alternatively, if the waves are out of step, they may cancel each other out (destructive interference). In this way, Barad not only allows for multiple possible outcomes but also that the prospect of high probability events occurring can be reduced through the interference effects encountered.

Barad’s performative approach has an appeal if we are to move beyond the traditional concepts of agency which tend to express behaviours in more purposive terms such as enabling, constraining or resisting. Some agencies may be purposive, intentional and deliberative, but not all need to be. It is the conjoint affect of the intra-action of agencies that is visible in the outcomes observed. Barad does not suggest that agency in the natural and the social worlds are necessarily equivalent, but that diffraction methods help to attend to the mutual entanglements that occur through their intra-action. Who or what is included (or excluded) in these entangled practices matters as it will influence the phenomena that result from changes in the system.

The value of taking a performative approach to agency is that it helps to move beyond the subject–object dualism and begins to recognise agency as an entanglement of agencies—placing ourselves in a more-than-human socio-hydrogeological world that is both objective and subjective, material and immaterial and where the past, present and future all exert influence. Our human actions/choices have power, but only through intra-action with other actors. As Mudliar and Koontz (2021) identify in the context of watersheds, negative manifestations of power may not always be overtly visible but may be signalled by the nonparticipation of some or the silence of nondominant actors. This silence does not mean that they are devoid of agency, merely that they respond through alternative means which may not be immediately apparent. Barad’s concept of a conjoint performative agency, set within a more-than-human world, begins a journey of exploring these alternative forms of agency.

## Conclusions

In this paper, I argue for a reframing of agency as emergent, distributed and enacted through intra-action, rather than as a fixed trait of individuals or objects. Crucially, taking a performative approach emphasises that agency does not require humanlike traits such as intent, but acknowledges that, where such traits are present, they will exert influence on emergent outcomes. Recognising how agency emerges from overlapping actions demonstrates that it is less about who, or what, exerts agency over others but how respective agencies coalesce and reinforce or cancel each other out. This respects Barad’s (2007) contention that agency is an emergent property that arises through a series of entanglements between human society, aquifers and the wider environment.

As the academy widely recognises, aquifers are complex actors combining material and immaterial dimensions which can vary across space and over time. It is the interplay of these dimensions that give aquifers their vitality and the ability to shape their environs and corresponding socio-hydrogeological systems. Aquifers are, though, just one actor amongst many in more-than-human landscapes. It is through the variety of intra-actions within a socio-hydrogeological system that various agencies emerge, entangle, have power, reconfigure and re-emerge. Acknowledging that aquifers enact agency as part of complex and adaptive socio-hydrogeological systems, encourages us to unpack the entangled relationships between aquifers, humans and other actors in the landscape. Doing so can offer novel insights regarding water-sensitive urban/rural development pathways that approaches which separate hydrogeological processes from the socio-economic may obscure.

Rethinking the role of aquifers and agency in socio-hydrogeological systems opens up novel interdisciplinary research agendas. As a relational and performative construct, agency is not ‘owned’ by any single actor but happens through intra-actions in practice, highlighting the significance of engaging a wide spectrum of perspectives when considering the stewardship of groundwater (de Bont and Börjeson 2024; Domínguez-Guzmán et al. 2023). As Barad identifies: ‘causal relations cannot be thought of as specific relations between isolated objects’ (Barad 2007). Whilst in this paper I do not engage with questions regarding the moral and ethical standing of aquifers, I do advocate for seeing aquifers as subjects not objects. As such, we must consider the contribution of non-human actors to agency as equivalent to that of human actors, although this may be differentially enacted. In doing so, we might begin to visualise agency as part of a lively, generative dance between multiple partners which varies over space and time. By foregrounding an aquifer’s material and immaterial vitality, its temporal legacies, and its capacity to shape socio-political outcomes, we develop new insights on future development pathways.

However, if we wish to follow these agendas, we may first need to reflect on the ontologies of own approaches and to reconsider how, as a hydrogeological community and as a wider society, we engage with aquifers and delineate them. As Robert MacFarlane (2025) submits: ‘to go unheard is not to be speechless. No landscape speaks with a single tongue’. Our challenge is to embrace a new socio-hydrogeological dialogue and to listen to the aquifer as part of reflexive intra-actions.
